# Digital quantum simulators in a scalable architecture of hybrid spin-photon qubits

**DOI:** 10.1038/srep16036

**Published:** 2015-11-13

**Authors:** Alessandro Chiesa, Paolo Santini, Dario Gerace, James Raftery, Andrew A. Houck, Stefano Carretta

**Affiliations:** 1 Dipartimento di Fisica e Scienze della Terra, Università di Parma, I-43124 Parma, Italy; 2 Dipartimento di Fisica, Università di Pavia, via Bassi 6, I-27100 Pavia, Italy; 3 Department of Electrical Engineering, Princeton University, Princeton, New Jersey 08544, USA

## Abstract

Resolving quantum many-body problems represents one of the greatest challenges in physics and physical chemistry, due to the prohibitively large computational resources that would be required by using classical computers. A solution has been foreseen by directly simulating the time evolution through sequences of quantum gates applied to arrays of qubits, i.e. by implementing a *digital quantum simulator*. Superconducting circuits and resonators are emerging as an extremely promising platform for quantum computation architectures, but a digital quantum simulator proposal that is straightforwardly scalable, universal, and realizable with state-of-the-art technology is presently lacking. Here we propose a viable scheme to implement a universal quantum simulator with hybrid spin-photon qubits in an array of superconducting resonators, which is intrinsically scalable and allows for local control. As representative examples we consider the transverse-field Ising model, a spin-1 Hamiltonian, and the two-dimensional Hubbard model and we numerically simulate the scheme by including the main sources of decoherence.

There is a large number of problems that are well known to be hardly tractable with standard computational approaches and resources, mainly due to the many-body nature of strongly correlated many particle systems. To overcome this limitation, the idea of a quantum simulator was originally proposed by Feynman^1^: any arbitrary complex quantum system could in fact be simulated by another quantum system mimicking its dynamical evolution, but under the experimenter control. This idea was later refined and mathematically formalized in quantum information perspectives by Lloyd^2^.

Over the past twenty years, different approaches have been proposed to realize quantum simulators of the most relevant models in condensed matter physics, quantum field theories, and quantum chemistry^3^. Most efficient protocols have been proposed and experimentally realized with trapped ions^4^,^5^. Generally speaking, quantum simulators can be broadly classified into two main categories: in *digital simulators* the state of the target system is encoded in qubits and its Trotter-decomposed time evolution is implemented by a sequence of elementary quantum gates^2^, whereas in *analog simulators* a certain quantum system directly emulates another one. Digital architectures are usually able to simulate broad classes of Hamiltonians, whereas analog ones are restricted to specific target Hamiltonians. For a recent review on these different approaches, we refer to ref. 3 and references therein.

Lately, superconducting circuits and resonators have emerged as an extremely promising platform for quantum information and quantum simulation architectures^6^,^7^,^8^,^9^,^10^,^11^,^12^. The first and unique theoretical proposal for a general-purpose digital simulator has been put forward only very recently^8^. In this proposal qubits encoded in transmons are dispersively coupled through a photon mode of a single resonator, and such coupling is externally tuned by controlling the transmon energies. However, the reported fidelities and the intrinsic serial nature of this setup (i.e., the need of addressing each pair of qubits sequentially), may hinder the scalability to a sizeable number of qubits. In addition, superconducting units are not ideal for encoding qubits owing to their relatively short coherence times. Indeed, spin-ensembles^13^,^14^,^15^ or even photons^16^,^17^ have been proposed as memories to temporarily store the state of superconducting computational qubits.

Here we consider an array of superconducting resonators as the main technological platform, on which hybrid spin-photon qubits are defined by introducing strongly coupled spin ensembles (SEs) in each resonator^18^,^19^. One- and two-qubit quantum gates can be implemented by individually and independently tuning the resonators modes through external magnetic fields. This setup can realize a universal digital quantum simulator, whose scalability to a large array is naturally fulfilled by the inherent definition of the single qubits, represented by each coupled SE-resonator device. The possibility to perform a large number of two-qubit gates in parallel makes the manipulation of such large arrays much faster than in a serial implementation, thus making the simulation of complex target Hamiltonians possible in practice.

A novelty of the present proposal is that ensembles of effective *S* = 1 spins are used in the hybrid encoding, which allows to exploit the mobility of photons across different resonators to perform two-qubit gates between physically distant qubits. This is done much more efficiently than by the straightforward approach of moving the states of the two qubits close to each other by sequences of SWAP gates, and makes the class of Hamiltonians which can be realistically addressed much larger. Long-distance operations arise whenever mapping the target system of the simulation onto the register implies two-body terms between distant qubits. Besides the obvious case of Hamiltonians with long-range interactions, this occurs with any two-dimensional model mapped onto a linear register, or with models containing *N*-body terms, including the many-spin terms which implement the antisymmetric nature of fermion wavefunctions.

The time evolution of a generic Hamiltonian is decomposed into a sequence of local unitary operators, which can be implemented by means of elementary single- and two-qubits gates. Then we combine the elementary gates of our setup in order to mimic the dynamics of spin and Hubbard-like Hamiltonians for fermions. We explicitly report our results for the digital quantum simulation of the transverse-field Ising model on 3 qubits, the tunneling dynamics of a spin one in a rhombic crystal field and the Hubbard Hamiltonian. We use a time-dependent Hamiltonian for this hardware including the effects of decoherence in a Lindblad formalism, thus performing extensive numerical experiments on our specific device, directly showing the feasibility of the proposed digital quantum simulation.

## Results

### A scalable architecture for quantum simulation

**Figure 1:**
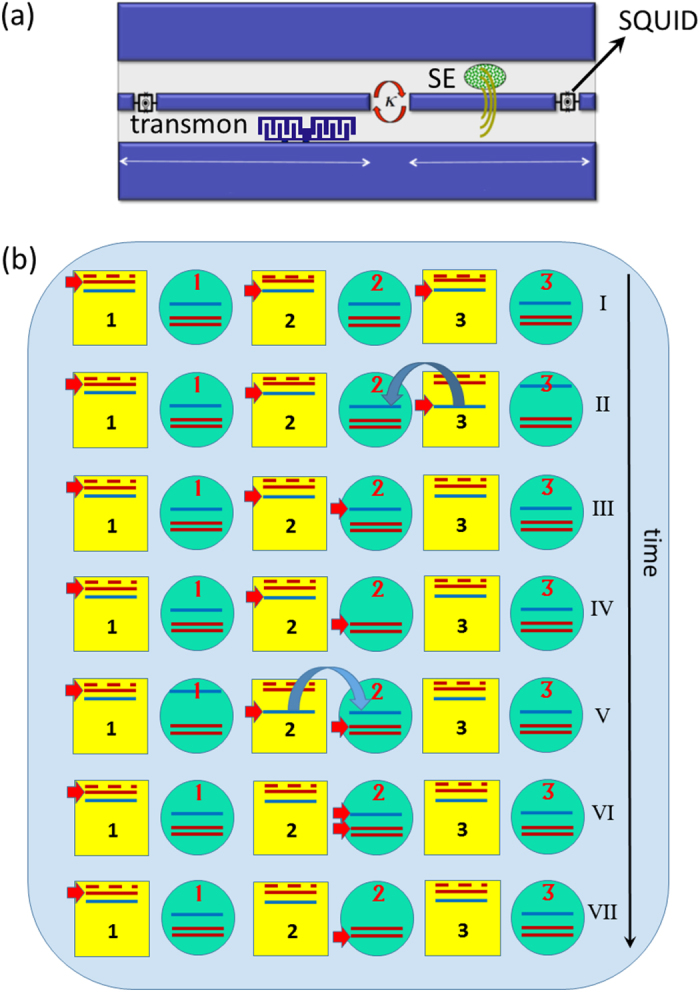
(**a**) Elementary unit of the scalable setup, consisting of an *auxiliary* and a *logical* resonator. The latter includes an ensemble of *S* = 1 spins, placed at the antinodes of the magnetic field (rotational lines) of the cavity mode. The *auxiliary* resonator contains a nonlinear element (transmon) coupled to the electric field of the fundamental mode. (**b**) Detailed sequence of time steps required to produce controlled-*φ* two-qubit gate between qubits *μ* = 2 and *μ* = 3 (see Methods for details). *Logical* cavities are represented by square boxes, whereas *auxiliary* resonators are depicted as circular boxes. Blue lines represent photon frequencies in the idle configuration (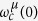 in the *logical* and 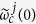 in the *auxiliary* cavities). The transmon (Ω_01_ and Ω_12_) and spin (*ω*_−1_, continuous, and *ω*_1_, dashed) transition energies are indicated by red lines. (I) qubits are initially into state 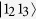, with the excitations (red arrows) stored into the photonic degrees of freedom (blue lines); (II) *logical* cavity 3 is brought into resonance with the *auxiliary* resonator *j* = 2, thus (III) bringing the photon to the auxiliary cavity. In the meantime *auxiliary* resonator 3 is detuned from the others to avoid unwanted photon hoppings. In (IV) the photon is absorbed by the transmon 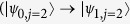 transition). The same hopping process (V) is repeated for the photon originally in cavity 2, which is brought to the *auxiliary* resonator (VI) and then absorbed and emitted by the transmon 
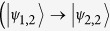
 transition) in a semi-resonant Rabi process (VII). The procedure is then repeated to bring photons back to *logical* cavities 2 and 3, leading the state back to 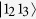 with an additional phase *φ* acquired during the semi-resonant process.

The proposed quantum simulator is schematically shown in Fig. 1. It consists of a one- or two-dimensional (1D or 2D) lattice of superconducting resonators where hybrid spin-photon qubits are defined. We notice that large arrays of such resonators have already been shown experimentally^7^,^20^. In this schematic implementation, qubits are encoded within square boxes. Each box represents a coplanar resonator containing an ensemble of (effective) *S* = 1 spins, whose collective excitations correspond to the transitions from the *m* = 0 single-spin ground state to the *m* = ±1 excited states, and can be modeled by two independent harmonic oscillators. Red lines represent the transition energies (continuous *m* = −1, dashed *m* = 1 transitions, respectively), while the blue line indicates the resonator frequency. This can be varied within a nanosecond time-scale by means of SQUID devices properly connected to the resonator^21^,^22^,^23^, in order to match the spin transition frequencies. In the hybrid qubit encoding, a dual-rail representation of the logical units is introduced where the 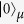 and 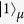 states of qubit *μ* are defined in the single-excitation subspace of each resonator. The logical state 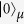 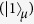 corresponds to zero (one) photons and a single (zero) quantum in the *m* = −1 oscillator in cavity *μ*. This encoding has been introduced in previous works^18^,^19^, and it is detailed in Methods for completeness. The *m* = 1 oscillator represents an auxiliary degree of freedom that is exploited to store the photonic component of the qubit, if needed (e.g., to perform two-qubit gates between distant qubits, see Methods).

The basic unit of the scalable array is represented by a pair of qubits connected through an interposed auxiliary resonator containing a superconducting transmon device (circular box), which is employed to perform two-qubit gates. It should be emphasized that this nonlinear superconducting element is not used to encode information, and *it is left in its ground state always except during the implementation of the two-qubit gates*. Consequently, its possibly short coherence times affect the quantum simulation only marginally. Other types of superconductor based qubits^24^, such as flux^25^ or Xmon^26^ qubits, can be exploited as well. Here we focus on transmon qubits^27^, which are commonly used thanks to their low sensitivity to charge noise.

In the following, we shall refer to the square boxes as the *logical* cavities labelled with Greek letters, while the circular ones are the *auxiliary* cavities labeled by Latin letters. Photon hopping between neighboring resonators is allowed by capacitive coupling. Formally, such a complex system can be described by the total Hamiltonian


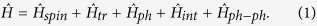


The first term describes the SEs as independent harmonic oscillators^28^ (*ħ* ≡ 1):


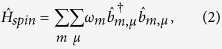


where 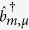 creates a spin excitation in level *m* = ±1 of resonator *μ*. The transmons are treated as effective three-level systems, with transition energies Ω_01_ and Ω_12_, and described by


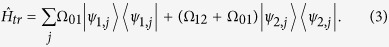


The time-dependent photonic term is entirely responsible for the manipulation of the qubits. It can be expressed as:


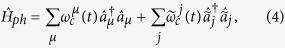


where 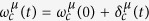 and a similar expression holds for 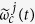; 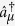 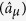 creates (destroys) a single photon in the *logical* resonator *μ*, while 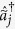 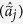 creates (destroys) a single photon in the *auxiliary* cavity *j*. Hereafter, we will use the interaction picture, with 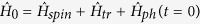. Hence, within the rotating-wave approximation the spin-photon and transmon-photon coupling Hamiltonian takes the form:


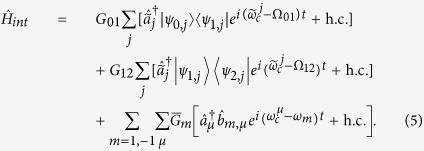


Here, the coupling constants 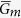 for the SE are enhanced with respect to their single-spin counterparts by a factor 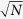, *N* being the number of spins in the SE^29^.

Finally, the last term in Eq. (1) describes the photon-hopping processes induced by the capacitive coupling between the modes in neighboring cavities^20^:


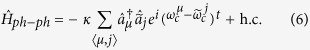


Single- and two-qubit gates are efficiently implemented by tuning individual resonator modes, as shown in previous works^18^,^19^. Arbitrary single-qubit rotations within the Bloch sphere as well as controlled-phase (C*φ*) gates can be realized (see Methods for a summary).

The present setup offers two remarkable benefits: the first is that using the hybrid encoding with an ensemble of effective *S* = 1 spins ensures the possibility of implementing C*φ* gates between distant qubits, with no need of performing highly demanding and error-prone sequences of SWAP gates. This is done by bringing the photon components of the two qubits into neighboring *logical* resonators by a series of hopping processes (see Methods for details). Transferring the photons with no corruption and without perturbing the qubits encoded in the interposed *logical* cavities is made possible by temporarily storing the photon component of these interposed qubits into the *m* = 1 spin oscillator.

In addition, quantum simulations can be performed in parallel to a large degree, with resulting reduction of simulation times. This is made possible by the definitions of the single qubits, represented by each coupled SE-resonator device, and by the local control of each *logical* or *auxiliary* resonator. Non-overlapping parts of the register can then be manipulated in parallel. For instance, in simulating a Heisenberg chain of *N* spins *s* = 1/2, the *N* two-qubits evolutions which appear at each time-step in the Trotter decomposition are performed first simultaneously on all *N*/2 “even” bonds and then simultaneously on the remaining *N*/2 “odd” bonds. Thus the simulation time of each Trotter step does not increase with *N*.

### Numerical experiments

While it is obvious that a universal quantum computer can be used in principle to simulate any Hamiltonian, the actual feasibility of such simulations needs to be quantitatively assessed by testing whether the complex sequences of gates needed are robust with respect to errors due to decoherence. Here we numerically solve the density matrix master equation for the model in Eq. (1) with the inclusion of the main decoherence processes, i.e., photon loss and dephasing of the transmons^19^ (see Methods for details).

In the following, we will consider the *fidelity*


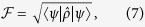


as a valuable figure of merit for the target Hamiltonians to be simulated, where 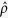 is the final density matrix and 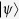 the target state. For the simulations shown in the following, we have chosen these operational parameters: *ω*_1_/2*π* = 37 GHz, *ω*_−1_/2*π* = 35 GHz, *ω*_*c*_(0)/2*π* = 31 GHz, 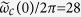 GHz and Ω_01_/2*π* = 21.7 GHz, Ω_12_/2*π* = 19.6 GHz (see the level scheme inside each cavity in Fig. 1). We also assume realistic values of the SE-resonator 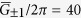 MHz, transmon-resonator *G*_01_/2*π* = 30 MHz, *G*_12_/2*π* = 40 MHz and photon-photon *κ*/2*π* = 30 MHz couplings, respectively^20^,^30^. The transmon parameters correspond to a ratio between Josephson and charge energies *E*_*J*_/*E*_*C*_ = 25^24^. In this regime the dephasing time 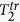 exceeds several *μs* while keeping a 10% anharmonicity. The two chosen spin gaps can easily be achieved with several diluted magnetic ions possessing a *S* > 1/2 ground multiplet, just by applying a small magnetic field along a properly chosen direction. We have chosen resonator frequencies *ω*_*c*_ and 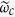 larger than usual experiments (e.g., twice the typical frequencies reported in ref. 30) since this helps improving the maximal fidelity of gates. However, we emphasize that the results do not qualitatively depend on these specific numbers. Indeed, high fidelities are also obtained by using resonator frequencies smaller than in ref. 30 (see Table 1).

#### Digital simulation of spin Hamiltonians

Since most Hamiltonians of physical interest can be written as the sum of *L* local terms, our quantum computing architecture can be employed to efficiently simulate the time-evolution induced by any target Hamiltonian of the type 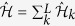. The system dynamics can be approximated by a sequence of unitary operators according to the Trotter-Suzuki formula (*ħ* = 1):


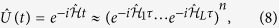


where *τ* = *t*/*n* and the total *digital* error of the present approximation can be made as small as desired by choosing *n* sufficiently large^2^. In this way the simulation reduces to the sequential implementation of local unitaries, each one corresponding to a small time interval *t*/*n*. The set of local unitary operators can be implemented by a proper sequence of single- and two-qubit gates.

The mapping of *s* = 1/2 models onto an array of qubits is straightforward. We consider here two kinds of significant local terms in the target Hamiltonian, namely one- 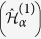 and two-body 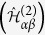 terms, with *α*, *β* = *x*, *y*, *z*. The unitary time evolution corresponding to one-body terms 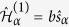 is directly implemented by single-qubit rotations 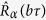. Conversely, two-body terms describe a generic spin-spin interaction of the form 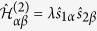, for any choice of *α*, *β* = *x*, *y*, *z*. The evolution operator, 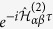, can be decomposed as^31^


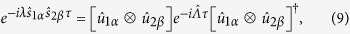


with 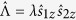, 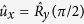, 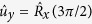, 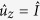. The Ising evolution operator, 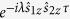, can be obtained starting from the two-qubit C*φ* gate and exploiting the identity (apart from an overall phase)


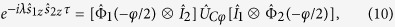


where *φ* = *λτ*. Here 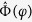 is a phase gate (see Methods). The time required and the fidelity for the simulation of each term of a generic spin Hamiltonian are calculated by using a Lindblad master equation formalism and are listed in Table 1. We notice that the predicted fidelities are very high, even after the inclusion of realistic values for the main decoherence channels, especially for the photon loss rate Γ_*μ*_, which is related to the resonators quality factor (*Q*) by 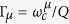. The high fidelity obtained for these elementary steps allows us to combine many of them to simulate multi-spin models.

**Figure 2:**
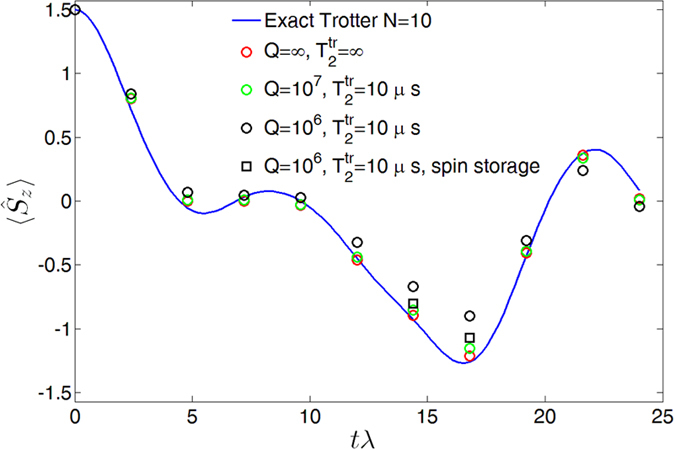
Oscillations of the magnetization in the transverse-field Ising model. The simulation is performed on a chain of 3 qubits, in the case *b* = *λ*/2. The plot reports the expectation value of the total magnetization 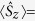 tr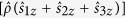 on the final state of the system, simulated for different values of the resonator quality factor (points) and compared with the exact evolution (line).

As a prototypical example we report the digital quantum simulation of the transverse field Ising model (TIM) on a chain of 3 qubits:


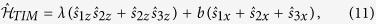


where 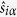 are spin-1/2 operators. Figure 2 shows the oscillations of the magnetization, Tr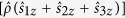, for a spin system initialized in a ferromagnetic configuration. Here 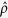 is the three-qubit density matrix obtained at the end of the *n* = 10 Trotter steps of the simulation. The exact Trotter evolution (continuous line) is compared to the simulated one (points). In particular, red circles represent the ideal evolution, without including any source of decoherence. Errors are, in that case, only due to a non-ideal implementation of the quantum gates (see discussion below). Conversely, green and black circles are calculated including the most important decoherence channels, namely photon loss (timescale 1/Γ_*μ*_) and pure dephasing of the transmon (timescale 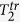). It turns out that photon loss is the most important environmental source of errors^19^, while 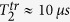
32 is sufficient to obtain high fidelities at the end of the simulation. Indeed, the transmon is only excited during the implementation of two-qubit gates. The simulation has been performed for different values of the resonators quality factor. By decreasing *Q* the average fidelity decreases from 96.5% (infinite *Q*) to 94.6% (*Q* = 10^7^) and 84.6% (*Q* = 10^6^). For high but realistic^33^ values of *Q* = 10^7^ the calculated points are close to the ones obtained in the ideal case (with infinite *Q*): in that case the gating errors still dominate the dynamics. Finally, by exploiting the auxiliary *m* = 1 oscillator to store the photon component of the hybrid qubits when these are idle, the effects of photon loss are reduced and the fidelity significantly increases. The improvement is evident in Fig. 2, by comparing black circular and square points; the final fidelity raises from 84.6% to 92% thanks to this storage. We stress again that the simulation time of each Trotter step does not increase for larger systems containing more than 3 spins. Indeed, even if more gates are needed, these can be applied in parallel to the whole array, independently of the system size. Below we shall also discuss the extension to a larger number of qubits.

The simulation of Hamiltonians involving *S* > 1/2 spin ensembles can be performed by encoding the state of each spin-*S* onto that of 2*S* qubits. As an explicit example, we consider a chain of *S* = 1 spins, labelled *S*_*i*_, with nearest-neighbor exchange interactions and single-spin crystal-field anisotropy, described by the Hamiltonian


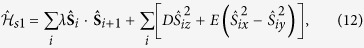


which reduces to the paradigmatic Haldane case for *D* = *E* = 0 and λ > 0. By rewriting each spin-1 operator as the sum of two spin-1/2 ones 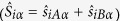, 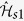 can be mapped onto a *s* = 1/2 Hamiltonian, 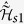, with twice the number of spins. Indeed, if each A-B pair of qubits is initialized into a state with total spin equal to one, the dynamics of 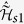 coincides with that of 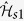 and can be simulated along the lines traced above. A proof-of-principle experiment, which could be implemented even by the non-scalable single-resonator setup described in ref. 19 would be the simulation of a single spin *S* = 1 experiencing tunneling of the magnetization. In this simple case we find (apart from a constant term):


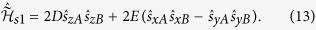


**Figure 3:**
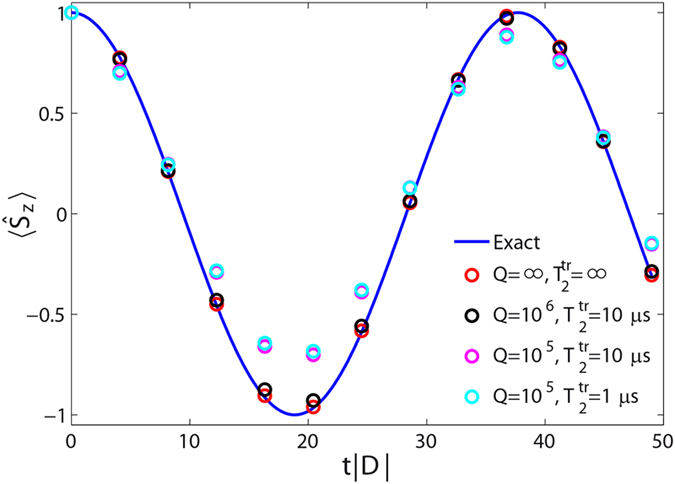
Tunneling of the magnetization in a spin-1 system. Line: exact time evolution of 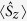 for a single *S* = 1 spin with |*D*/*E*| = 12, after Eq. (13). As it is well known, the system oscillates between states with opposite magnetization due to quantum tunneling across the anisotropy barrier. Points: digital quantum simulation obtained by the time evolution of two hybrid qubits for different values of the resonator quality factor, *Q*, and of the transmon coherence time, 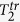, respectively.

Figure 3 reports the comparison between the exact and the simulated evolution of the magnetization, assuming *D* < 0 and |*D*/*E*| = 12, for different values of *Q* and 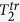. Interestingly, quantum oscillations of 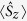 are well captured by the simulation even for *Q* = 10^5^, and the fidelity is practically unaffected by a reduction of transmon coherence time to 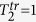 *μ*s.

The simulation of many-spin models with *S* > 1 typically requires two-qubit gates involving non-nearest-neighbor qubits. These can be handled with no need of SWAP gates as outlined in Methods.

#### Digital simulation of Fermi-Hubbard models

The numerical simulation of many-body fermionic systems is a notoriously difficult problem in theoretical condensed matter. In particular, quantum Monte Carlo algorithms usually fail due to the so-called sign-problem^34^. Our digital quantum simulator setup enables to efficiently compute the quantum dynamics of interacting fermions, even on an arbitrary two-dimensional lattice. Although we focus on the paradigmatic Fermi-Hubbard Hamiltonian, the proposed scheme can be generalized to the quantum simulation of several other fermionic models, such as the Anderson impurity model.

The target Hamiltonian describing a two-dimensional *N* × *M* lattice of Wannier orbitals is


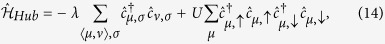


where 〈*μ*,*ν*〉 are nearest neighbors (*ν* = *μ* ± 1, *ν* = *μ* ± *M*) and 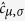 are fermionic operators. In order to simulate this Hamiltonian with our setup, we exploit the Jordan-Wigner transformation to map fermion operators 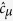 onto spin ones 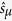
35,36,37. However, if such a transformation is applied to the Hubbard model (14) in more than one dimension, the hopping (first) term results into XY spin couplings whose sign depends on the parity of the number of occupied states that are between *μ* and *ν* in the chosen ordering of the Wannier orbitals^38^. This aspect makes the simulation of a fermionic system much more demanding than any typical spin system, because the resulting effective spin Hamiltonian contains many-spin terms. To illustrate how we address this key issue, here we consider the simpler case of the hopping of spinless fermions on a lattice (the general case of interacting spin fermions is discussed in Methods). The target Hamiltonian can be mapped into the following spin model:


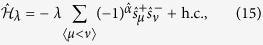


where 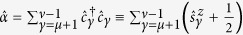. We simulate this *n*-body interaction by taking care of the state-dependent phase, similarly to refs 39,40. The sign factor in (15) is obtained by performing a conditional evolution of the qubits interposed between the specifically addressed sites, *μ* and *ν*, depending on the state of *μ*. This corresponds to a series of controlled-Z (CZ) gates between qubit *μ* and each of the qubits *γ* interposed between *μ* and *ν*. Hence, the sequence of gates to be implemented at each Trotter step is the following:


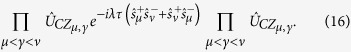


**Figure 4:**
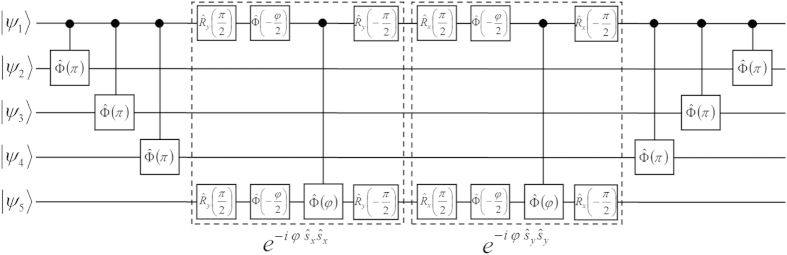
Quantum circuit description for the simulation of the hopping part of the Fermi-Hubbard model on a two-dimensional lattice. Here we explicitly show the case of 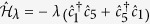, with *φ* = 2*λτ*. 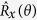 and 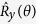 indicate single-qubit rotations about *x* or *y* axis of the Bloch sphere, while 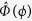 is the single-qubit phase gate.

For instance, in Fig. 4 we show the quantum circuit for the implementation of 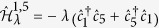: controlled-phase gates (with *φ* = *π*) between qubit 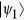 and each of the qubits interposed between 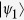 and 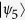, namely 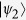, 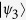 and 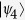, are sequentially performed before and after the central block (dashed boxes), which implements the XY evolution: 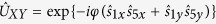. The latter consists of two controlled-*φ* gates (with *φ* = 2*λτ*), preceded and followed by proper single-qubit rotations, implementing respectively 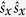 and 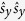 terms of the interaction, as schematically explained in Fig. 4. By exploiting the high mobility of the photons entering into the hybrid encoding, Hamiltonian terms involving distant qubits can be simulated straightforwardly. In fact, this is a remarkable advantage with respect to alternative solid-state platforms for quantum information processing. We stress that, in spite of the increment in the number of gates required to address the sign issue, a large number of hopping terms can still be implemented in parallel.

## Discussion

We have proposed a digital quantum simulator based on hybrid spin-photon qubits, encoded in an array of superconducting resonators strongly coupled to spin ensembles. Within this quantum computing architecture, quantum gates are implemented by a single operational tool, namely by tuning the resonators frequencies. We have shown the feasibility of the scheme with state-of the-art superconducting arrays technology, which allows the high fidelity simulation of a large class of multi-qubits spin and fermionic models. To test our predictions, we have performed numerical simulations of the master equation for the system density matrix, including the most important decoherence channels such as photon loss and pure-dephasing of the transmon involved in two-qubit entangling gates.

### Sources of errors

We analyze here the sources of error that affect the quantum simulation, and point out possible solutions. Three main simulation errors can be found: digital errors (arising from the Trotter-Suzuki approximation), gating errors (due to imperfect implementation of the desired unitaries), and decoherence errors (due to the interaction of the quantum simulator with the environment). While digital errors can obviously be reduced by increasing the number of Trotter steps or by using higher-order decompositions, gating errors are accumulated by repeating a large number of quantum operations. Similarly, the interaction of the system with the environment becomes much more pronounced if the simulation time increases.

As far as decoherence mechanisms are concerned, we first notice that the present setup *limits the role of the transmon*, which is not involved in the definition of the qubits. All transmons are kept in their ground states apart from the specific transmons involved in two-qubit gates, which are excited only for a short time. Thus, typical state-of-the-art technology, which ensures transmon dephasing times of the order of tens of microseconds, is sufficient to obtain high fidelity quantum simulations of relatively large systems. Coherence times of single spins are so long that their effect on quantum simulations can be disregarded. However, a potential drawback of spin ensembles is the presence of disorder which spreads the transition frequencies within the ensemble (inhomogeneous broadening). This eventually results in an irreversible population leakage from the superradiant mode (our logical 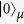, strongly coupled to the resonator) into dark modes out of the computational basis. In the absence of cavity-spin coupling, this leakage effect depends crucially on the width Δ of the distribution in the emitter’s bare frequencies *ρ*(*ω*), and takes place on a timescale ~1/Δ. However, a strong spin-cavity coupling provides a protection mechanism^41^, by inducing an energy gap between the superradiant and the dark modes^42^. If this gap is large enough, the system is efficiently protected from decoherence and the excitation can be stored in 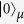 for times much longer than 1/Δ. This mechanism, which has been experimentally demonstrated in resonant conditions^43^, also acts in the dispersive regime, provided that the SE-cavity detuning (*δ*) is not too large, i.e. 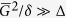. A detailed treatment of how to process hybrid qubits in a cavity protected regime is beyond the aim of this work and will be given elsewhere. An alternative possibility is that of using refocusing techniques^15^,^44^ in order to increase the coherence time of the inhomogeneously broadened spin ensemble.

Photon loss represents the main source of decoherence in our hybrid dual-rail encoding. Its effect monotonically increases with the overall computational time, since both idle and manipulated qubits are influenced by it. We stress that the proposed platform allows us to manipulate simultaneously non-overlapping parts of the register, thus drastically reducing the overall computation time and decoherence-induced errors with respect to a serial implementation. Indeed, a pessimistic estimate of the decoherence error 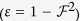 on *N* qubits subject to photon loss is given by 
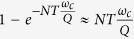
, where *T* is the total time of the simulation. This is obtained by considering the probability for the system prepared in the state with the maximum number of photons 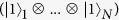 to be still in the same state after time *T*. For simple Hamiltonians (e.g. the TIM or the Heisenberg model), in a serial implementation *T* scales with *N*, whereas in a parallel scheme *T* is independent of *N*. For example, the three-spins transverse Ising model reported above can immediately be extended to simulate longer spin chains, by addressing simultaneously first the “odd-bonded” and then the “even-bonded” qubits. Hence, the parallel implementation proposed here leads to a gain in the fidelity scaling as 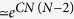, with respect to an analogous serial scheme. This makes the present architecture very competitive, in view of scaling it to a relatively large array.

In this parallel implementation, for simulation times much smaller than the characteristic photon loss damping time 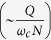, errors are mainly due to gate imperfections. Using the numbers reported in the third column 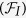 of Table 1, we can heuristically estimate the number of gates allowed by the proposed platform. For simplicity, we follow ref. 8 and assume single-gate errors as independent and add them. As a threshold, we require the overall fidelity after the implementation of the full sequence to be above 90%. This would allow us to perform more than 1000 single-qubit rotations or ~120 controlled-Z two-qubit gates. For instance, in the extension of the simulation of the transverse field Ising model to *N* = 10 qubits, the estimated gating error *ε*_*g*_ for each Trotter step is still very small, below 0.02 (corresponding to a fidelity of 99%). In the case of the more demanding *N* = 10 Heisenberg model we find *ε*_*g*_ = 0.07.

We note that gating errors are mainly due to the relatively small difference 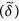 between the photon frequency and transmonic gaps in the auxiliary cavities, which induces a residual interaction that is never completely switched off. This leads to a leakage of a fraction 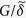 of the wave-function, which decreases the fidelity. Here we use the tunability of the resonator frequency as the only tool to process quantum information, but the flux control of the Josephson energy of the transmons^27^,^45^ can also be exploited to increase the detuning, thus leading to even larger fidelities. This additional degree of freedom would in turn allow us to employ larger values of the transmon-resonator couplings (commonly reported in literature), thus reducing the time required to implement two-qubit gates and hence the effect of decoherence. To keep the experimental demonstration of the proposed scheme as easy as possible, in the above simulations we have employed the tunability of the resonators as the only manipulation tool, using parameters which are a trade-off between the two effects of reducing the gating time and increasing the leakage.

We finally remark that quantum error correction (QEC) would also represent a powerful tool to improve the performance of the digital simulator. QEC schemes can be embedded in the present setup. For instance we could introduce ancillae qubits to implement the three qubit bit-flip and phase-flip codes^46^. These consist of single qubit rotations, two qubit gates between each ancilla and the logical qubit and a three qubit Toffoli gate (or equivalently a controlled-controlled-Z gate). In a one-dimensional logical array, the ancillae can be placed just above and below each logical qubit, connected to a common auxiliary resonator. In this way the ancillae can directly interact with the logical qubit, allowing us to implement two-qubit gates between them. The controlled-controlled-Z gate can be obtained without decomposing it into a more demanding sequence of two qubit gates, in a way similar to that proposed for the controlled-Z gate, by exploiting the fourth level of the transmon to induce a 3-step Rabi flop. The detailed description of this scheme is beyond the scope of this work and will be given elsewhere.

### Two-dimensional arrays

**Figure 5:**
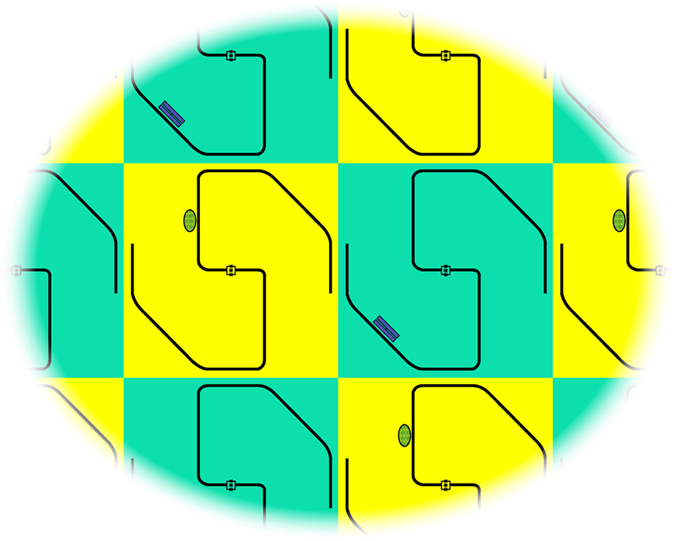
Schematic representation of a two-dimensional implementation of the digital quantum simulator. Dark lines show superconducting coplanar resonators routed such that each resonator is coupled to four adjacent resonators. Yellow boxes indicate *logical* resonators containing ensembles of S = 1 spins near the magnetic field antinodes, while green boxes indicate *auxiliary* resonators containing transmons near voltage antinodes. Flux biasing of the resonator SQUIDs could be accomplished using microwave lines placed on another layer.

While any model can be implemented onto a one-dimensional register (e.g., the one schematically illustrated in Fig. 1) at the cost of requiring long-range two-qubit gates, it is clear that a register topology directly mimicking the target Hamiltonian would greatly reduce the simulation effort. In particular, there are several important Hamiltonians defined on two-dimensional lattices whose simulation would greatly benefit from a two-dimensional register. Here, we point out that our scheme is straightforwardly usable on such a register, but its experimental realization necessarily requires the implementation of two sub-lattices of cavities, alternatively coupled to spin and transmon qubits, respectively. Fortunately, resonator arrays with complex network topologies are realistically possible, already, as each cavity can easily couple to multiple other resonators. Figure 5 displays the schematic drawing of a potential two-dimensional layout showing how such sub-lattices could feasibly realize a two-dimensional simulator. From a technological point of view, we notice that similar lattices with transmon qubits have been fabricated with more than 200 coupled cavities^7^. While local tuning in such a lattice would require local flux bias on a separate layer, this need for local control lines applies to any adjustable quantum simulator. On the other hand, we notice that a recent technology has shown promising results to bring flux lines to the interior part of a lattice made of a small number of nodes, e.g. by using Aluminum airbridge crossovers to route microwave signals into a target resonator^47^.

### Summary

In conclusion, the proposed setup exploits the best characteristics of distinct physical systems: the long coherence times of the spins, which can encode quantum information and protect it from decoherence, and the mobility of photons entering this hybrid encoding of qubits. In the end, this allows to realize long-range two-body interactions between distant qubits without the need for much more demanding SWAP gates. Moreover, on-site tunability and scalability make this architecture extremely appealing and competitive with respect to alternative proposals, either based on superconducting arrays or on different technologies.

## Methods

### Hybrid dual-rail encoding

We consider a coplanar waveguide resonator containing a single photon in a mode of frequency *ω*_*c*_, and an ensemble of 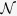 non-interacting and equally oriented *s* = 1 spins. In the low-excitation regime, the SE can be modeled by two independent harmonic oscillators, related to two different magnetic-dipole transitions from the *m* = 0 ground state of the single spin, to the *m* = −1 and *m* = 1 states, with excitation frequencies *ω*_−1_ and *ω*_1_. This can be achieved by properly choosing a system with easy-plane magnetic anisotropy, which provides a zero-field splitting between the *m* = 0 ground state and the excited *m* = ±1 doublet, and in the presence of a small static magnetic field. We suppose to initialize the system by preparing each spin in its ground state: 
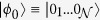
.

If the resonator frequency is tuned to match the spin gap *ω*_1_, the SE can absorb the photon and collectively evolve into the state 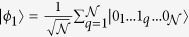. Transitions between 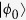 and 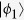 are described (in the limit of low number of excitations) by the bosonic operators 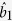 and 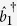, where 
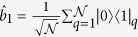
 and 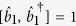
13,29. Conversely, if the resonator frequency is tuned to *ω*_−1_, the SE can evolve into the state 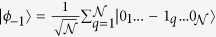, the transition being described by the operators 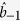 and 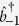, where 
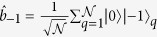
.

Within the single-excitation subspace of the system formed by the cavity mode and the SE, we introduce the hybrid dual-rail encoding of the qubit *μ*:


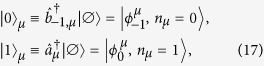


where 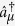 is the photon creation operator and 
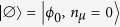
 is the vacuum state.

### Single- and two-qubit gates

#### Single-qubit rotations

Resonant processes involving the absorption (emission) of the photons entering the hybrid encoding in (Eq. 17) are exploited to perform one- and two-qubit gates. These processes are induced by “shift pulses”, in which the frequency of cavity *μ* is varied by a quantity 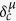 for a suitable amount of time. In the idle mode, the photon frequencies are largely detuned from the spin energy gaps, and 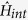 is ineffective. In addition, the modes 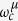 and 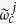 of neighboring cavities are far-detuned and the effect of 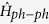 is negligible. Single-qubit gates can thus be performed independently on each qubit, which can be individually addressed.

Off-resonance pulses are employed to obtain a rotation by an arbitrary angle about the *z* axis of the Bloch sphere. These induce a phase difference between the 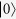 and 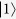 states of the hybrid qubits (Eq. 17) and performs the well-known phase gate:


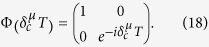


where we have assumed step-like pulses of amplitude 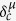 and duration *T*.

Conversely, resonant pulses are employed to transfer the excitation between SEs and resonators. This produces a generic rotation in the *x*-*y* plane of the Bloch sphere:


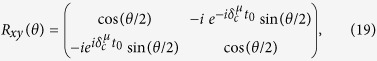


with 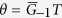. By properly tuning the initial time we can obtain rotations about *x* 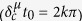 or *y* 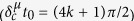 axis, while the pulse duration controls the rotation angle. See ref. 19 for a detailed derivation.

#### Controlled-phase gate

The Controlled-phase (C*φ*) two-qubit gate is represented by the matrix:


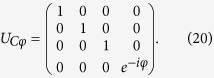


It can be implemented by means of two-step semi-resonant Rabi oscillations of the transmon state between 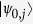 and 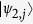. We describe here the C*φ* multi-step pulse sequence on two qubits initialized in the state 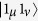, as schematically shown in Fig. 1(b) for *μ* = 2, *ν* = 3 and *j* = 2:

1. The first step corresponds to the hopping of the photon from *logical* cavity 3 to the *auxiliary* resonator 2 (interposed between qubits 2 and 3), by means of a *π*-pulse that brings the two cavities into resonance.
2. As a second step, the frequency of resonator *μ* = 2 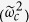 is tuned to Ω_01_ by means of a *π*-pulse, which transfers the excitation to the intermediate level 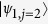 of the transmon.
3. A *π*-pulse is exploited to induce the hopping of a second photon from *logical* cavity 2 to the *auxiliary* resonator.
4. Then, a semi-resonant process (during which the resonator is detuned from the transmon gap by a small amount *δ*_12_) is exploited to induce an arbitrary phase on the 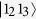 component of the wavefunction^16^. A pulse of duration 
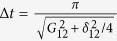
, where 
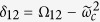
 is the detuning between the resonator mode and the 
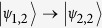
 transition of the transmon, adds a phase 
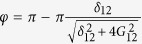
 to the system wavefunction.
5. Finally, the repetition of the first three steps brings the state back to 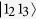, with an overall phase *φ*. By properly setting the delay between the two *π* pulses corresponding to the previous steps (or by performing single-qubit phase shifts), the associated absorption and emission processes yield a zero additional phase.

Conversely, the other basis states do not acquire any phase, as required for the C*φ* gate, due to the absence of at least one of the two photons (see ref. 19). For *δ*_12_ = 0 we obtain the usual full Rabi process, which implements a Controlled-Z (CZ).

The setup is simplified with respect to our previous proposal^18^, as each resonator contains a single photonic mode.

It is also important to note that here we are using an ensemble of effective spins *S* = 1 as this ensures the possibility of implementing Controlled-phase gates between distant qubits, with no need of performing highly demanding and error-prone sequences of two-qubit SWAP gates. Long-distance two-qubit interactions are a key-resource for the digital simulation of many interesting physical Hamiltonians. They appear each time that a multi-dimensional target system is mapped onto a linear chain of qubits or in models with *N*-body terms. Among these, as discussed in the main text, a particular interest is assumed by problems involving interacting fermions in two or higher spatial dimensions, which are often intractable for classical computers. For instance, solving the two-dimensional Hubbard model is considered by many as the ultimate goal of the theory of strongly correlated systems. In these cases the Jordan-Wigner mapping induces many-spin interactions^40^ which can be handled as outlined in Fig. 4, provided the ability to efficiently implementing long-range two-qubit couplings. These are obtained by bringing the photon components of the two qubits into neighboring *logical* resonators by a series of hoppings. The operations outlined in Fig. 1(b) are then performed to implement a C*φ* gate between neighboring qubits, and the photon components are finally brought back to the starting position by reverting the series of hoppings. The photons can be transferred with negligible leakage and without perturbing the interposed qubits by temporarily storing the photon component of these qubits into the *m* = 1 spin oscillator. We stress that a large number of these long-range two-qubit gates can be implemented in parallel in the actual setup.

### Density Matrix Master Equation

The time evolution of the system density matrix 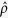 is described within a Markovian approximation and a Lindblad-type dynamics, with the Liouville-von Neumann equation of motion^48^:


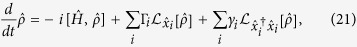


being Γ_*j*_ and *γ*_*j*_ respectively the damping and pure-dephasing rates of the field 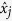. The Lindblad term for an arbitrary operator, 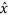, is given by


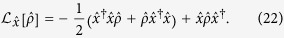


If the operator 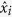 destroys an excitation in the system, terms like 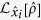 account for energy losses, while pure dephasing processes are described by 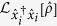. We note^19^ that the former ones provide the most important contribution for photons^49^ (with 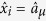, 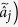, while the latter are very important for the transmons (
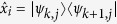
, *k* = 0, 1). We represent each field as a matrix in the Fock-states basis, and truncate it at a number of total excitations previously checked for convergence. The total Hamiltonian, Eq. (1), and the density matrix master equation of the whole system, Eq. (21), are built by tensor products of these operators. Then, the equation of motion for 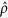 is numerically integrated, in the interaction picture, by using a standard Runge-Kutta approximation.

#### Interacting spin fermions

To extend the quantum simulation of two-dimensional Hubbard models to the case of fermionic systems with spin, we need to encode each fermion operator into a pair of qubits, corresponding to spin up and spin down. To achieve this, we exploit a generalization of the Jordan-Wigner transformation^50^. For this mapping we need to introduce two different spin 1/2 operators 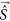 and 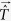, with 
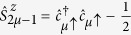
 and 
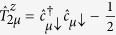
, describing respectively odd and even qubits (ordered by rows in the two-dimensional lattice).


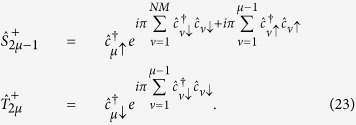


It can be shown that these operators satisfy the usual angular momentum commutator algebra, and that 
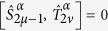
. We assume that the fermion variables are ordered by rows in the Hamiltonian. The efficiency of the scheme would be increased by using a 2-dimensional setup consisting of *N* rows and 2*M* columns. We can write the Hubbard Hamiltonian in terms of the spin variables introduced above


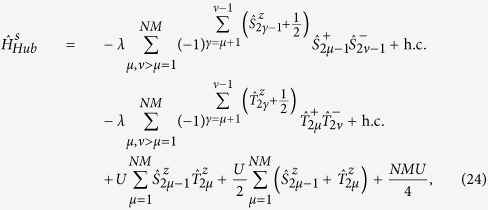


where *μ* and *ν* are nearest neighbors on the two-dimensional fermionic lattice, such that *ν* = *μ* + 1 (horizontal neighbors) or *ν* = *μ* + *M* (vertical neighbors) with the present labeling. Odd (even) qubits encode spin up (spin down) variables. Since the hopping term does not act if 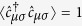 (i.e. 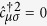), we can start directly with *γ* = *μ* + 1, and the exponential in expressions like 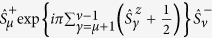 can be factorized. We note that in the case of horizontal neighbors the phase factor cancels out and that in 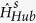 do not appear terms 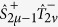, as we are not considering spin-flip processes.

To simulate such evolution we can proceed in a way analogous to the spinless case. Here, however, two different series of *CZ*_*μ*,*γ*_ should be carried out, depending if we are considering the hopping of spin ↑ or spin ↓ fermions. The former involves only odd values of *γ*, the second only even. Notice that, in a 2-dimensional register, we need to transfer photons to implement 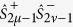 or 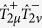 each time we have to couple a pair of fermions belonging to the same row (due to the alternating ↑-↓ mapping), but in that case ’ 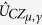 is not required. The term ’ 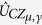, needed to correct the *sign problem*, is necessary only if *ν* = *μ* + *M* (no photon transfer in that case is needed).

## Additional Information

**How to cite this article**: Chiesa, A. *et al.* Digital quantum simulators in a scalable architecture of hybrid spin-photon qubits. *Sci. Rep.* **5**, 16036; doi: 10.1038/srep16036 (2015).

## Figures and Tables

**Table 1:** Simulation of the elementary terms of the Hamiltonian.

	Time	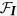	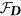	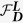
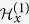	6.4 ns	99.99%	99.94%	99.79%
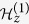	0.5 ns	99.99%	99.98%	99.90%
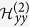	85.8 ns	99.87%	99.24%	98.96%
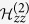	61 ns	99.91%	99.45%	99.20%
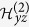	85.8 ns	99.79%	99.13%	98.87%

Fidelity and time required to simulate the elementary terms of the Spin Hamiltonian. The fidelity has been calculated by assuming a random initial state. The second and third column show a comparison between the ideal fidelity (calculated in the absence of decoherence) and the real one (calculated assuming a Lindblad dynamics, with *Q* = 10^6^ and 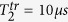). The implemented evolution is 
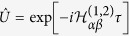
, with *bτ* = *λτ* = *π*/2. The last column reports the fidelities corresponding to a setup operating at lower frequencies, *ω*_1_/2*π* = 16.05 GHz, *ω*_−1_/2*π* = 15.7 GHz, *ω*_*c*_(0)/2*π* = 14 GHz, 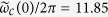 GHz and Ω_01_/2*π* = 9.2 GHz, Ω_12_/2*π* = 8.3 GHz.
